# A Green Approach to Modify Surface Properties of Polyurethane Foam for Enhanced Oil Absorption

**DOI:** 10.3390/polym12091883

**Published:** 2020-08-21

**Authors:** Zhi Chien Ng, Rosyiela Azwa Roslan, Woei Jye Lau, Mehmet Gürsoy, Mustafa Karaman, Nora Jullok, Ahmad Fauzi Ismail

**Affiliations:** 1 Advanced Membrane Technology Research Centre (AMTEC), School of Chemical and Energy Engineering, Universiti Teknologi Malaysia, Johor Bahru 81310, Johor, Malaysia; christine_zcng@yahoo.com (Z.C.N.); rosyielaazwa.roslan@yahoo.com (R.A.R.); afauzi@utm.my (A.F.I.); 2 Department of Chemical Engineering, Konya Technical University, Konya 42075, Turkey; mgursoy@ktun.edu.tr (M.G.); mkaraman@ktun.edu.tr (M.K.); 3 Centre of Excellence for Biomass Utilization, Universiti Malaysia Perlis (UniMAP), Kompleks Pusat Pengajian Jejawi 3, Jejawi 02600, Perlis, Malaysia; norajullok@unimap.edu.my

**Keywords:** PU foam, CVD, surface modification, oil absorption, water

## Abstract

The non-selective property of conventional polyurethane (PU) foam tends to lower its oil absorption efficiency. To address this issue, we modified the surface properties of PU foam using a rapid solvent-free surface functionalization approach based on the chemical vapor deposition (CVD) method to establish an extremely thin yet uniform coating layer to improve foam performance. The PU foam was respectively functionalized using different monomers, i.e., perfluorodecyl acrylate (PFDA), 2,2,3,4,4,4-hexafluorobutyl acrylate (HFBA), and hexamethyldisiloxane (HMDSO), and the effect of deposition times (1, 5 and 10 min) on the properties of foam was investigated. The results showed that all the modified foams demonstrated a much higher water contact angle (i.e., greater hydrophobicity) and greater absorption capacities compared to the control PU foam. This is due to the presence of specific functional groups, e.g., fluorine (F) and silane (Si) in the modified PU foams. Of all, the PU/PHFBA_i_ foam exhibited the highest absorption capacities, recording 66.68, 58.15, 53.70, and 58.38 g/g for chloroform, acetone, cyclohexane, and edible oil, respectively. These values were 39.19–119.31% higher than that of control foam. The promising performance of the PU/PHFBA_i_ foam is due to the improved surface hydrophobicity attributed to the original perfluoroalkyl moieties of the HFBA monomer. The PU/PHFBA_i_ foam also demonstrated a much more stable absorption performance compared to the control foam when both samples were reused for up to 10 cycles. This clearly indicates the positive impact of the proposed functionalization method in improving PU properties for oil absorption processes.

## 1. Introduction

The spills of oil in seas/rivers and on land could pose serious threats to environments owing to the presence of a wide range of hazardous and volatile organic compounds. Low-cost yet effective approaches for oil spill removals are thus very important. Several representative methods that are widely used for oil spill treatment are oil dispersant, oil gelling agent, and oil sorbent [1,2]. Although each method has its own advantages and disadvantages, the use of sorbents outperforms the other two methods because it can be employed to remove oil from the sea/river.

Polymeric foams made of polyurethane (PU) perhaps are the most popular organic sorbents for oil spill treatment due to their low cost and rapid absorption rate [1]. Even though they are good oil sorbents, PU foams still suffer from a low oil absorption capacity. The existence of ether-ester-, carbamate-, and amide-groups in the PU foam has caused it to absorb not only oil molecules but also water, which reduces its performance efficiency [3].

As reported in the literature, the performance of PU foams could be enhanced by subjecting them to various surface modifications, including spray coating [4], grafting [3,5], plasma-induced graft co-polymerization [6], and hydrothermal processing [7]. Nevertheless, it must be pointed out that most of the surface modification approaches are time-consuming and require the use of solvents/hazardous chemicals. For instance, the polyethyleneimine (PEI)/reduced graphene oxide (RGO)-coated PU foam synthesized by Periasamy et al. [7] required 24 h for the PEI to be cross-linked with ethylenediaminetetraacetic acid (EDTA) and another 3-h heating at 95 °C to reduce GO. Prior to the surface modification, the PU foam was soaked in a PEI solution followed by adding mixtures of EDTA, GO, and 1-ethyl-3-(3-dimethylaminopropyl)carbodiimide (EDC). Another main concern of this work is the use of expensive nanomaterials for surface modification which set a barrier for large-scale production, even though such nanomaterial-modified foams tended to offer enhanced absorption capacities.

Li et al. [3], on the other hand, performed a 6-h chemical reaction to graft the surface of PU foam with oleophilic monomers such as lauryl methacrylate (LMA), butyl methacrylate (BMA), and styrene (St) followed by thorough ethanol rinsing to remove impurities. The hexadcene (HD)/LMA-coated PU foam synthesized by Keshawy et al. [8], meanwhile, required 12-h treatment in an oven at 100 °C after the PU foam was soaked in the HD/LMA mixture several times.

Compared to the above-mentioned surface modification techniques, the chemical vapor deposition (CVD) method is considered more advantageous for surface modification owing to several main reasons, i.e., the fast reaction time (ranging from seconds to several minutes), the fact that it is environmentally friendly (without involving organic/hazardous solvents), the elimination of post-treatment (i.e., ethanol rinsing after modification), and highly controllable layer thickness (in nm-scale), as well as uniform coating properties [9,10,11]. Among the various CVD processes, plasma-enhanced CVD (PECVD) and initiated CVD (iCVD) are the most common and mature types of CVD for the fabrication of polymeric thin films.

Both methods have their own pros and cons. For instance, polymeric films resulted from the iCVD are usually linearly structured and thus might not be durable in certain applications [12]. The ion bombardment during PECVD enables the production of highly stable crosslinked polymeric thin films, but excessive ion bombardment is likely to damage chemical structures of deposited polymeric films [13]. Hence, iCVD polymeric thin films usually show better functional group retention than those of PECVD thin films.

There are several studies conducted to compare the efficiency of both methods for a specific application [14,15]. In this work, we intend to employ both methods to functionalize the PU foam surfaces and compare the resultant modified foams, particularly for the oil absorption process. Three different hydrophobic monomers, i.e., perfluorodecyl acrylate (PFDA), 2,2,3,4,4,4-hexafluorobutyl acrylate (HFBA), and hexamethyldisiloxane (HMDSO), were utilized to functionalize the PU foams, and the effect of plasma deposition time (1, 5, and 10 min) was investigated on the absorption capacities of modified foams against different kinds of organic solvents and oil. The changes in the foam surface properties were further characterized by using a series of instruments including a contact angle goniometer, Fourier-transform Infrared spectroscope (FTIR), field emission scanning electron microscope (FESEM), and energy-dispersive X-ray spectroscope (EDX).

## 2. Materials and Methods

The PU foam used in this work was supplied locally by Preeco Engineering Sdn. Bhd (Selangor, Malaysia) (Density: 13 kg/m^3^, Size: 42 inch (W) × 75 inch (L) × 20 mm (T)). To modify the surface of foams based on CVD methods, three monomers, i.e., HFBA (95%), PFDA (97%), and HMDSO (≥98%), obtained from Sigma-Aldrich (St. Louis, MO, USA), were utilized. For the modification process via the iCVD method, initiator di-tert butyl peroxide (TBPO, 98%) purchased from Sigma-Aldrich (St. Louis, MO, USA) was required. Several kinds of organic solvents, including chloroform (≥99%) and acetone (≥99%) acquired from Merck (Kenilworth, NJ, USA), cyclohexane (≥99%) purchased from Fisher Scientific (Waltham, MA, USA), and edible vegetable oil (Type: Palm oil) obtained from a local supermarket (Johor, Malaysia) were used in this study. All chemicals/materials were used as received without any further modification and purification.

### 2.1. Surface Functionalization of Foam

In this work, two different functionalization techniques, i.e., PECVD and iCVD, were employed to modify the surface properties of foams, and each technique would deposit two kinds of polymers under varying deposition times (1, 5 and 10 min) onto the foams. PECVD thin film depositions were performed in a cylindrical Pyrex glass chamber with an inner diameter of 6 cm and length of 30 cm. The details of the design can be found elsewhere [16]. Foam samples were first placed into the chamber. The chamber with copper coil wound externally was then connected to a 13.56 MHz radio frequency plasma generator (MKS, Andover, MA, USA). A matching network was placed between the copper coil and the plasma generator to reduce the standing wave ratio. A rotary-vane vacuum pump (Edwards XDS-10, Burgess Hill, UK) was used to create a vacuum in the chamber. The pressure was continuously measured by a capacitance type pressure gauge (MKS, Baratron, Andover, MA, USA). The respective monomer (either HFBA or HMDSO) was then placed in stainless jars before being clamped to the manifold pipeline to feed into the chamber at room temperature. The flowrate of the monomer was precisely adjusted using a high-accuracy needle valve. The optimum deposition conditions for HFBA functionalization were as follows: 100 mtorr reactor pressure, 40 W plasma power, and 2 sccm monomer flowrate. Meanwhile, the optimum conditions for the HMDSO functionalization were reported at 75 mtorr reactor pressure, 70 W plasma power, and 1 sccm monomer flowrate. The functionalized foam samples are designated as PU/PHFBA and PU/PHMDSO, depending on the type of monomers used during PECVD process.

iCVD thin film depositions were performed in a custom-built stainless reactor, and its detailed description can be found elsewhere [17]. Foam samples were placed into the reactor bottom in which a heat exchanger was located on the backside of the reactor bottom. The temperature of the samples was kept at the desired value using a recirculating water chiller (Thermo Neslab, Waltham, MA, USA). The required activation energy to initiate polymerization was provided by a tungsten filament array (located 2.5 cm above the reactor bottom) which was heated to a set temperature. In order to monitor the filament temperature, a K-type thermocouple was attached to the filament array. A rotary-vane vacuum pump (Edwards XDS-10, Burgess Hill, UK) was used to create a vacuum in the reactor. The pressure was continuously monitored by a capacitance-type pressure gauge (MKS, Baratron, Andover, MA, USA). The desired pressures were set by placing a throttle valve between the reactor and the pump. The monomer (either HFBA or PFDA) together with the initiator (TBPO) placed in separate jars were then fed into the reactor in which their respective flowrate was adjusted with the help of needle valves. The initiator was fed into the reactor at room temperature while the temperatures of the monomer jars and manifold pipelines were kept constant at desired values using PID temperature controllers.

For iCVD HFBA deposition, the temperatures of the monomer jar and manifold pipeline were set constant at 30 °C and 40 °C, respectively. For PFDA deposition, the monomer jar and manifold pipeline were kept at 55 °C and 70 °C, respectively. The optimum deposition parameters for iCVD HFBA functionalization were found to be: 1100 mtorr chamber pressure, 200 °C filament temperature, 2 sccm monomer flowrate, and 2 sccm initiator flowrate. The optimum deposition parameters of iCVD PFDA functionalization meanwhile were reported at 150 mtorr chamber pressure, 280 °C filament temperature, 0.5 sccm monomer flowrate, and 0.8 sccm initiator flowrate. The functionalized foam samples are designated as PU/PHFBA_i_ and PU/PPFDA_i_, depending on the type of monomers used during the iCVD process.

### 2.2. Oil/organic Solvent Absorption

**Figure 1:**
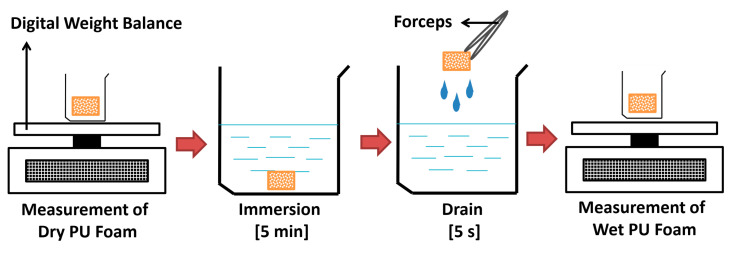
Schematic illustration of the procedure for oil/organic solvent absorption test.

The method used to measure the absorption capacity of PU foams was based on the ASTMF726-99-Standard Test Method for Sorbent Performance of Adsorbents. Figure 1 illustrates the procedure of conducting absorption tests for each type of PU foam. In brief, 250 mL of each type of oil/organic solvent sample (chloroform, acetone, cyclohexane, and edible oil) was separately poured into four beakers. The initial weight, also known as the dry weight, of the PU sample (dimension: 1 cm × 1 cm × 1 cm) was recorded prior to the immersion process. Thereafter, the dry PU foam was dropped into a respective beaker and immersed for 5 min. After 5 min, the foam was removed from the beaker and held for 5 s to drain off the excess or loosely adhered oil/organic solvent. Following this, the saturated foam was quickly transferred to a high precision digital balance (readability: Up to 0.001 g). The final weight, also known as the saturated weight, was then recorded. Equation (1) was employed to calculate the absorption capacity of each PU foam:

Oil sorption (g/g) = (S_t_ − S_0_)/S_0_ (1)

where S_0_ is the initial dry weight of the foam and S_t_ is the weight of foam together with the weight of oil/organic solvent absorbed.

### 2.3. Recyclability Test

A recyclability test was further conducted to investigate the reusability of the control and modified PU foams, whereby only one modified PU foam that generates the best absorption performance from Section 2.2 was selected for this evaluation. The protocol for the recyclability test was similar to the absorption test described in Section 2.2, except the test was repeated for 10 cycles using the same foam. After each absorption cycle, the foam was squeezed and wiped gently with tissue to remove the absorbed oil before proceeding to the next cycle.

### 2.4. Characterization

Prior to analysis, all samples were dried in an oven for at least 24 h at 40 °C. The surface chemistries of the control and modified PU foams were analyzed using Fourier transform infrared spectroscopy (FTIR) on a Perkin Elmer Frontier (Waltham, Massachusetts, USA). For FTIR, the sample was scanned under attenuated total reflectance (ATR) mode, with an average of 8 scans and wavenumber range of 650–4000 cm^−1^. Field emission scanning electron microscopy (FESEM) (Hitachi, SU8000, Tokyo, Japan) coupled with an energy dispersive x-ray spectrometer (EDX) (Oxford, Xmax-N 50 mm, Abingdon, UK) were used to analyze morphological and chemical changes in foams before and after surface modification. The sample was cut into small pieces before being adhered to sample stubs using copper tape. Prior to scanning, an ultrathin layer of platinum was sputter-coated onto the sample surface using a rotary pump coater (Quorum, Q150RS, Laughton, East Sussex, UK). The surface wettability of foams was investigated using the contact angle goniometer on Data physics OCA 15Pro via the sessile drop method. RO water was used as a probe liquid, and the size of each drop was fixed at 2 μL. For each foam sample, an average of 10 measurements was randomly taken to yield the result. Selected foams (i.e., control PU and PU/PHFBA_i_ with 5 min deposition time) were further assessed by respectively immersing them (dimension: 1 cm × 1 cm × 1 cm) in ~120 mL of pure RO or Rhodamine B fluorescent aqueous solution. The immersed foam samples were shined using a light-emitting diode (LED) lamp and the photographs were taken using a high-resolution camera. This test was conducted to reaffirm the hydrophobicity of modified PU foam. The porosity of foams was determined using the saturation method [18]. The dry weight of each PU foam (dimension: 1 cm × 1 cm × 1 cm) was first recorded prior to dropping the foam into a beaker containing 100 mL of edible oil. Then, the foam was pressed and released multiple times in the oil solution, forcing the oil to enter the foam until the foam was completely saturated. The foam was considered saturated when no air-bubble was observed in the foam and the weight of soaked foam remained constant. Equations (2)–(4) were then used to calculate the foam porosity, Φ.

S_b_ = S_s_ − S_d_ (2)

(3) $$V_{p}\;=\;S_{b}/\rho_{b}$$

(4) $$\mathrm{Porosity}\;(\Phi )=V_{p}/V_{t}$$

where S_b_ is the weight of oil absorbed, S_s_ is the weight of saturated foam, S_d_ is the dry weight of foam, V_p_ is the pore volume of foam, ρ_b_ is the density of edible oil (0.9092 g/cm^3^), and V_t_ is the bulk volume of the foam.

## 3. Results and Discussion

### 3.1. Characterization of Control and Modified PU Foams

#### 3.1.1. Surface Chemistry

**Figure 2:**
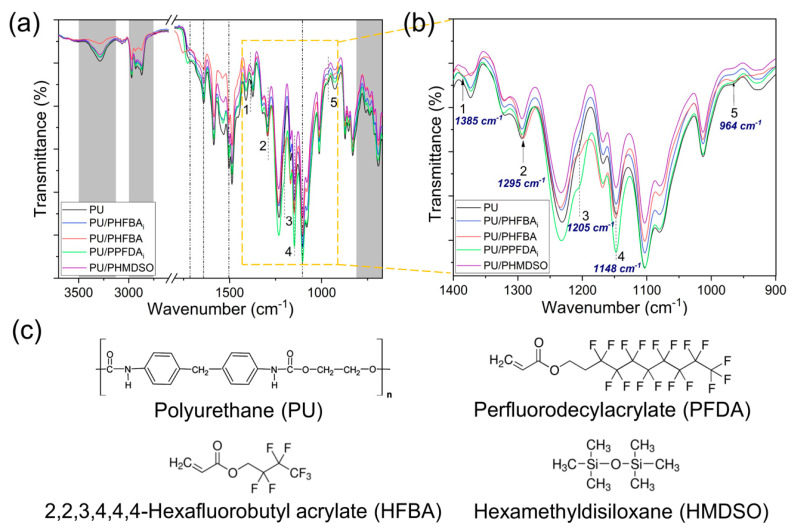
ATR-FTIR spectra of the control and modified PU foams (5 min deposition time) at spectra ranges of (**a**) 2600–3700 cm^−1^ and 670–1800 cm^−1^ and (**b**) 900–1400 cm^−1^, and (**c**) the chemical structures of PU foam and monomers.

The surface chemistry of the foam is substantially important during oil/solvent absorption as it plays a significant role in determining its absorption capacity. Figure 2a shows the ATR-FTIR spectra of PU foams without and with surface modification. The modified foams were prepared at a deposition time of 5 min. The typical characteristic peaks of PU including 3130–3500 cm^−1^ (N-H stretching), 2750–3000 cm^−1^ (-CH_2_ stretching), 1715 cm^−1^ (C=O stretching of ester), 1640 cm^−1^ (C=O stretching of urea), 1503 cm^−1^ (N-H deformation), 1103 cm^−1^ (C-O stretching) and 670–810 cm^−1^ (C-H deformation of aromatic ring) are observed on all the modified and control foam samples [19,20,21,22]. Compared to the control PU foam, the intensities of these peaks are reduced upon surface functionalization owing to the formation of a thin coating layer on the surface of the foam as a result of the CVD process.

Depending on the type of monomer used during CVD modification, the changes in the characteristic peaks of modified PU foam can be clearly seen in the wavenumber range of 900–1400 cm^−1^ as shown in Figure 2b. Compared to the control PU, both PU/PPFDA_i_ and PU/PHFBA show higher peak intensities at 1205 cm^−1^ (symmetric stretching of -CF_2_) and 1148 cm^−1^ (-CF_2_-CF_3_ end group) [23,24], indicating the PU surface is successfully functionalized. Characteristic peaks belonging to HFBA such as 1385 cm^−1^, 1295 cm^−1^, and 964 cm^−1^ correspond to C-H bending and asymmetric stretching of -CF_2_ and -CF vibration [23,25], respectively, are also observed on the PU/PHFBA. Nevertheless, it must be pointed out that these characteristics are not found in the PU/PHFBA_i_ that is also composed of HFBA but deposited using a different technique (i.e., iCVD). For the PU/PHFBA_i_, the changes in its peaks are more obvious (compared to the control PU) in the wavenumber of 3130–3500 cm^−1^, 2750–3000 cm^−1^, and 1640 cm^−1^. In other words, it can be said that the use of different CVD methods could result in different outcomes even though the same monomer was used. Compared to HFBA and PFDA, HMDSO is different as it does not possess fluorine (F) in its organic structure. Nonetheless, similar to PU/PHFBA_i_, the change in the peak of PU/PHMDSO was insignificant. The chemical structures of control PU foam together with three monomers used to functionalize the surface of PU foam are shown in Figure 2c. Changes in PU/PHFBA_i_ and PU/PHMDSO are almost undetectable because the concentrations of both Si and F are very low. Further EDX surface analysis reveals the low concentration of Si (0.13 at.%) and F (0.45 at.%) elements in both samples (Table 1).

Supplemental Figure S1 compares the impact of deposition time on the ATR-FTIR spectra of the PU foam modified with HFBA using the iCVD method. At 10 min deposition time, the intensity of several peaks found on the PU/PHFBA_i_ including 1385 cm^−1^, 1295 cm^−1^, 1205 cm^−1^, and 1148 cm^−1^ are seen to be slightly higher than the control PU. As shown in Supplemental Table S1, the atomic concentration of fluorine (F) increases with increasing coating time. Nevertheless, it is important to mention that the analysis performed using FTIR and EDX might not be sufficient to represent the functional groups of PU/PHFBA_i_ because they are not sensitive enough to determine the marginal change on the surface chemistry of modified PU owing to the extremely thin coating layer (in nanometers thick) formed on the PU foam during the CVD process.

#### 3.1.2. Structural Properties

**Figure 3:**
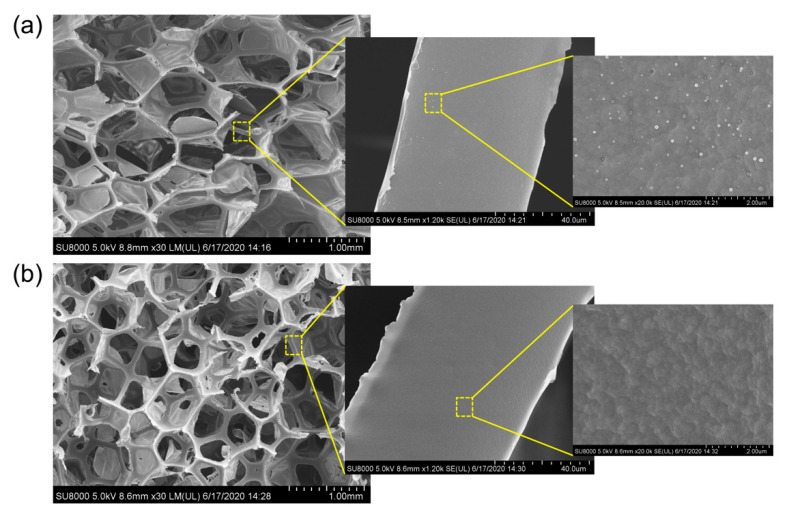
FESEM images of (**a**) control PU and (**b**) PU/PHFBA_i_ (5 min deposition time) viewed at increasing magnifications of ×30, ×1200, and ×20,000.

As reported in other studies [16,23], there is no significant difference in terms of the surface structure of the samples before and after CVD modification due to the extremely thin coating layer formed. Thus, only two samples (PU and PU/PHFBA_i_) were examined in this work using FESEM and the microscopical images are presented in Figure 3. The general appearance of the PU foam remains unchanged after the surface coating, suggesting that the coating layer is ultrathin and conformal. We can see that the PU/PHFBA_i_ foam exhibits a relatively smoother surface compared to the control PU foam when both samples are examined under higher magnification (×20,000). The small white dots on the surface of the control foam are the protrusions that are naturally found in PU foam [26,27]. The changes in the surface morphology can indicate the impact of CVD modification on the surface properties of foam at the nm level. Evidently, the porosities of all modified PU foams with the highest deposition time remain almost unchanged when compared to the control PU (see Table 2). Typically, increasing the deposition time of the CVD process corresponds to a thicker coating layer formed, but still the changes in coating layer thickness does not alter the foam porosity significantly.

#### 3.1.3. Surface Wettability

**Figure 4:**
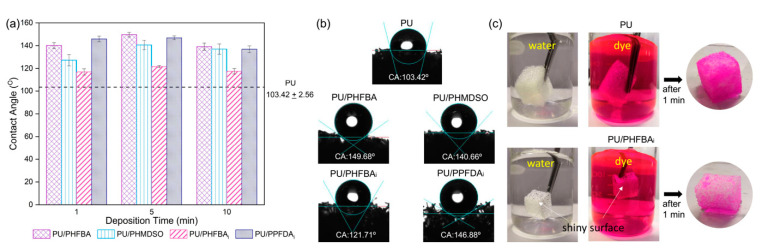
(**a**) The water contact angle of PU foams with and without surface functionalization, (**b**) cross-sectional images of a single water droplet on the surface of different PU foams, and (**c**) photographic images of PU and PU/PHFBA_i_ (5 min deposition time) in water and dye solution.

The effect of surface modification on the wettability of foams is further investigated and the results are shown in Figure 4. According to Law [28], a surface is considered hydrophilic when the static water contact angle (CA), *θ* is < 90° and hydrophobic when *θ* > 90°. Therefore, the high CA of PU (103.4°) as shown in Figure 4a implies that the PU is intrinsically hydrophobic. Upon surface modification, all the resultant foams show higher CA than the control PU, indicating that the hydrophobicity of foams is elevated. Hydrophobicity is critical in oil or organic solvent absorption processes as the greater the hydrophobicity of foam the higher its affinity towards oil or organic molecules.

In this study, the main elements that contribute to the improved surface hydrophobicity of PU foam are the F and Si functionality of the coated polymers. The presence of F is found to increase the CA of PU foam at a greater extent than Si in which the water CA levels of PU/PHFBA (139.16–149.68°) and PU/PPFDA_i_ (136.92–146.88°) are higher than the PU/PHMDSO (127.30–140.66°). The lower CA of PU/PHMDSO could be due to its extremely low Si concentration when compared to the F concentration found in PU/PHFBA and PU/PPFDA_i_, (see Table 1). When comparing the PU/PHFBA with the PU/PPFDA_i_ foam, it is found that the CA of the former is higher than the latter. The higher CA of PU/PHFBA foam is due to the existence of a branched fluorine-containing polymer. Such an observation is consistent with the findings of Zhang et al. [29] in which the ((CF_3_)_2_CF)(C_3_F_7_)CHOC(O)CH_2_=CH_2_ homopolymer is more hydrophobic compared to the C_6_F_13_(CF_3_)CHOC(O)CH=CH_2_ homopolymer. In addition to the impact of specific functional groups on the CA of foam, the change in the surface roughness of PU foam upon modification is also able to alter water CA. According to the Wenzel equation [30], a lower relative surface area yielded from a smooth surface could result in a slightly higher CA.

Figure 4b presents the cross-sectional images of a single water droplet on the surface of the control PU and PU foams modified at 5 min deposition time. As seen from the image, water CA decreases in the order of PU/PHFBA > PU/PPFDA_i_ > PU/PHMDSO > PU/PHFBA_i_ > PU. Even though PU is naturally hydrophobic, water droplets can still penetrate the foam due to its porous structure. The penetration of water into the porous PU foam is strictly inhibited after hydrophobic surface modification. Figure 4c compares the characteristics of PU/PHFBA_i_ foam (with the lowest CA among modified PU foams) with the control PU foam in water and dye solution. The shiny surface of the PU/PHFBA_i_ foam upon immersion in both solutions, resulted from the reflection of solutions surrounding the foam, indicates the reduced affinity of foam towards water molecules after the surface is hydrophobically tailored. Water tends to penetrate easier into the control PU foam, and because of this, it appears darker in color after 1-min absorption process.

### 3.2. Oil and Organic Solvent Absorption Capacity

**Figure 5:**
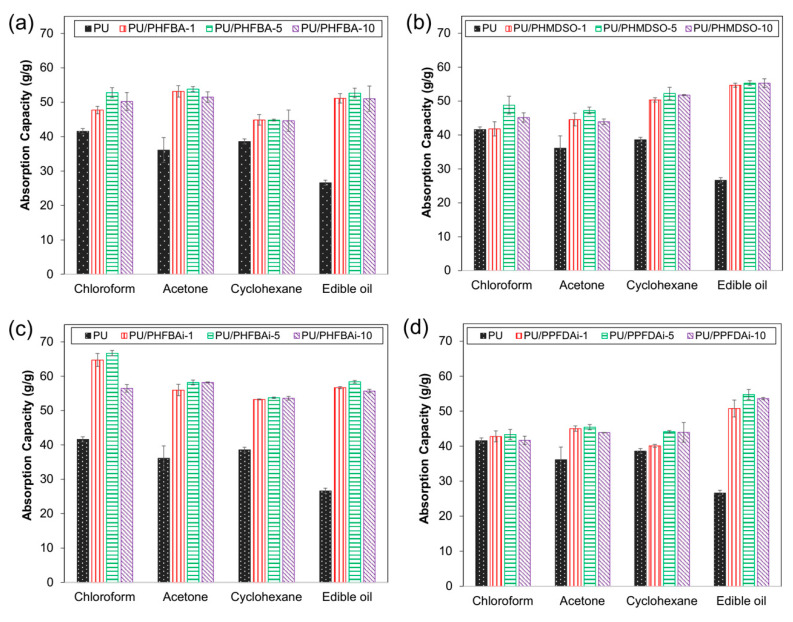
Oil or organic solvent absorption capacities of different types of PU foams: (**a**) PU/PHFBA, (**b**) PU/PHMDSO, (**c**) PU/PHFBA_i_, and (**d**) PU/PPFDA_i_.

The design of PU foam is responsible for its intrinsic absorptive property. Its 3D hierarchical porous structure allows all kinds of liquid to easily flow into it while its interconnected pores (see Figure 3) further promote liquid penetration via a capillary effect. Figure 5 shows the absorption capacity of control PU and modified PU foams towards four different samples. Prior to surface modification, PU foam exhibits higher absorption capacity towards hydrocarbon (i.e., chloroform, acetone, and cyclohexane) than edible oil. Such a phenomenon is ascribed to the lower viscosity of hydrocarbon (see Table 3) that allows it to diffuse at a faster rate into the PU foam than oil [8,18]. Because of this reason, PU foam swells rapidly upon contact with organic solvents.

The absorption capacity of PU foam is clearly improved after it is hydrophobically modified using the CVD method. Overall, the absorption capacity of foam increases in the order of PU < PU/PPFDA_i_ < PU/PHMDSO < PU/PHFBA < PU/PHFBA_i_. The hydrophobicity of fluorinated polymers can be affected by two factors, (i) the length of fluorocarbon chain and (ii) the degree of branching. Theoretically, a long fluorocarbon chain should equip polymer with better hydrophobicity, but this situation diverts with the increasing number of -CF_3_ exposed [29,31]. When the same quantity of PFDA and HFBA is used for polymerization, short-chain of HFBA tends to generate hyperbranched polymer with more -CF_3_ moieties compared to the long-chain PFDA. Consequently, the PU/PHFBA foam exhibits better oil and organic solvent absorption than the PU/PPFDA_i_ foam. Meanwhile, the low absorption capacity of PU/PHMDSO foam could possibly be due to the hydrolyzed Si entities that compromise material hydrophobicity and reduce its absorption performance [32].

Compared to the PECVD technique used to develop PU/PHFBA foam, iCVD is found to be more effective in producing promising foam (i.e., PU/PHFBA_i_) for oil and organic solvent absorption. At the optimum deposition time (5 min), the PU/PHFBA_i_ foam achieves significantly higher oil/solvent absorption than that of PU/PHFBA foam for four types of samples tested. It shows absorption rates of 66.68, 58.15, 53.70, and 58.38 g/g for chloroform, acetone, cyclohexane, and edible oil, respectively. The PU/PHFBA foam, meanwhile, only demonstrates 52.77, 53.83, 44.80, and 52.66 g/g, respectively. The lower capacity of the PU/PHFBA foam could be attributed to the higher degree of polymerization. Unlike the iCVD modification, the plasma discharge of the PECVD method could lead to an unavoidable loss of functional groups [23].

Table 4 further compares the oil and organic solvent absorption capacity of the PU/PHFBA_i_ foam prepared in this study with other modified PU foams reported in the literature. As can be seen, our PU/PHFBA_i_ foam not only shows a promising absorption capacity compared to other modified foams but also takes significantly less time to produce, i.e., 5 min.

### 3.3. Recyclability Test

**Figure 6:**
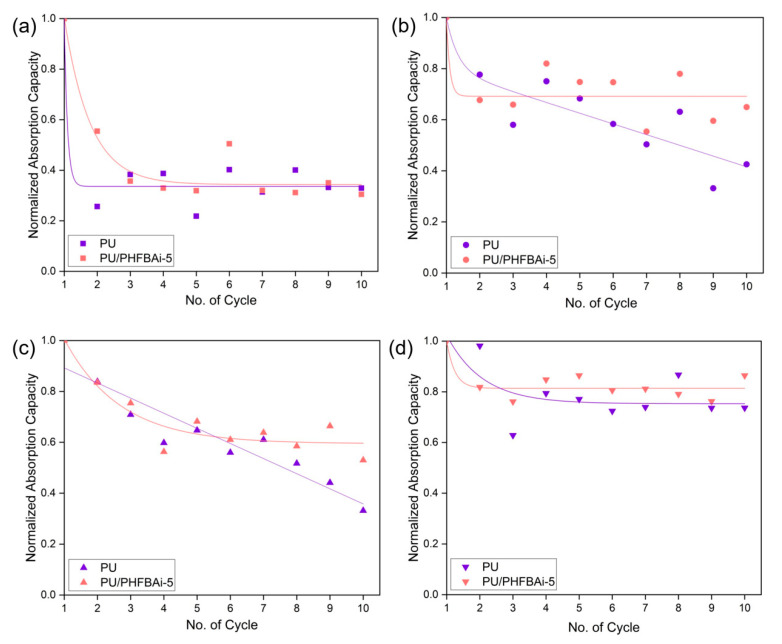
Recyclability of control PU and PU/PHFBA_i_-5 foam towards absorbing (**a**) chloroform, (**b**) acetone, (**c**) cyclohexane, and (**d**) edible oil for up to 10 cycles. The absorption capacity for each cycle is normalized by the initial weight of oil or organic solvent absorbed.

The recyclability test is a necessary test to determine the feasibility of sorbents for practical applications. Figure 6 shows the normalized absorption capacity of control PU and PU/PHFBA_i_ foam for up to 10 cycles. Overall, it can be seen that the PU/PHFBA_i_ foam demonstrates a much more stable performance compared to the control PU foam, except for the case of chloroform absorption that shows slightly better performance. The drop in the absorption capacity of the control PU and PU/PHFBA_i_ foam after the 1st cycle suggests that some of the oil or solvent remains in the foam sample after the squeezing process, i.e., a process to remove oil or solvent from the foam before re-absorption takes place.

**Figure 7:**
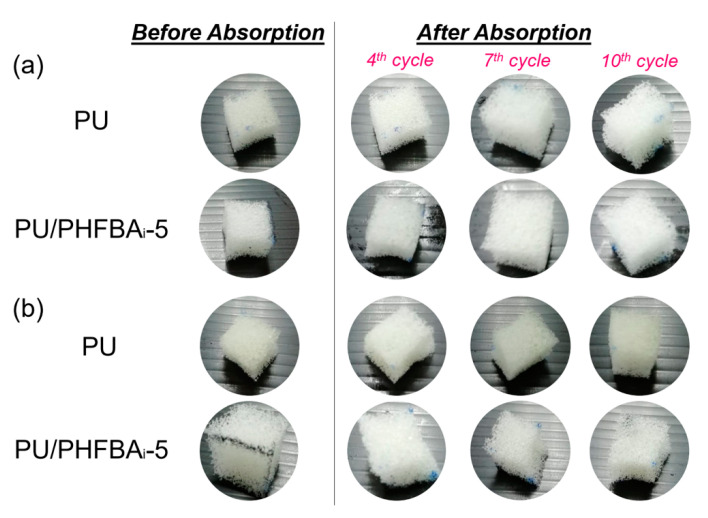
Photographic images of squeezed PU and PU/PHFBA_i_-5 foams before and after absorption against (**a**) cyclohexane (the most viscous solutions among tested solvents) and (**b**) edible oil.

As mentioned in Section 3.2, the absorption behaviours of foam, such as its capacity and rate, are highly related to the viscosity of the oil or organic solvents to be absorbed. Owing to the high diffusion rate of less viscous solution, we observed from the experiment that all PU foams (with or without modification) swell rapidly upon contacting with an organic solvent. It is observed that the PU foams are reversible (in terms of shape) for the first few cycles of testing, but experienced severe deformation after 7th cycles (see Figure 7). Even though the foam structure (shape) is distorted, the modified foam (PU/PHFBA_i_) still shows a higher absorption rate compared to the control PU foam after 10 cycles of absorption. In addition, its absorption capacity also remains stable between the 4th and 10th cycles. This could be due to the presence of specific functional groups on the modified PU foam that improve its surface chemistry, minimizing the drop in the absorption performance.

## 4. Conclusions

In this work, a rapid surface modification process based on the chemical vapor deposition (CVD) method was employed to functionalize the surface properties of polyurethane (PU) foam for enhanced absorption capacity. The PU foam was respectively functionalized using different monomers, i.e., perfluorodecyl acrylate (PFDA), 2,2,3,4,4,4-hexafluorobutyl acrylate (HFBA) and hexamethyldisiloxane (HMDSO), and the effect of the deposition times of the respective monomer (1, 5 and 10 min) on the surface properties of the foam and its absorption performance was investigated. As revealed from the results, the hydrophobicity of all modified foams was substantially improved after surface functionalization due to the presence of specific functional groups, namely fluorine (F) and silane (Si), thereby leading to their enhanced absorption capacities when compared to the control PU foams. This method was proven to be effective in modifying the chemical properties of foam without disturbing its physical properties. FESEM images also showed that the formed coating layer on the modified PU foams was extremely thin, with no significant effect on foam porosity examined under high magnification. The formation of a thin and uniform coating layer is the main advantage of the CVD method. Among all foams, PU/PHFBA_i_ foam was reported to demonstrate the highest absorption capacities against the oil and organic solvent samples tested, followed by PU/PHFBA, PU/PHMDSO, PU/PPFDA_i_, and PU foam. The PU/PHFBA_i_ foam showed absorption rates of 66.68, 58.15, 53.70, and 58.38 g/g for chloroform, acetone, cyclohexane, and edible oil, respectively. Its value was 60.33%, 61.04%, 39.19%, and 119.31%, respectively, higher than that of the control PU foam. The promising performance of the PU/PHFBA_i_ foam is due to its improved surface hydrophobicity attributed to the retained perfluoroalkyl moieties of HFBA deposited via the iCVD method. It must also be pointed out that the PU/PHFBA_i_ foam demonstrated a much more stable performance compared to the control PU foam when both samples were reused for up to 10 cycles. This clearly reveals the reusability of our modified PU foams. Our work demonstrated a rapid solvent-free surface functionalization method that can be utilized to enhance the PU foam properties for oil absorption processes.

## Figures and Tables

**Table 1:** EDX analysis of the control and modified PU foams (5 min deposition time).

Element	Atomic Concentration (at%)				
	PU	PU/PHFBA_i_	PU/PHFBA	PU/PPFDA_i_	PU/PHMDSO
C	81.23	82.42	79.23	76.18	82.10
O	18.77	17.13	18.22	14.95	17.77
F	-	0.45	2.54	8.87	-
Si	-	-	-	-	0.13

**Table 2:** The porosity of the control PU and modified PU foams (10 min deposition time).

Foam	Porosity
PU	0.95 ± 0.08
PU/PHFBA_i_	0.95 ± 0.02
PU/PHFBA	0.94 ± 0.00
PU/PPFDA_i_	0.91 ± 0.02
PU/PHMDSO	0.93 ± 0.06

**Table 3:** Properties of oil and organic solvents used.

Sample	Viscosity, μ (mPa·s)
^1^ Edible oil	69.1
^2^ Chloroform	0.537
^2^ Acetone	0.306
^2^ Cyclohexane	0.894

^1^ Data obtained for 25 °C from “Advances in Material Sciences and Engineering”, Awang M., Emaniam S. and Yusof F., Springer [33]. ^2^ Data obtained for 25 °C from “CRC Handbook of Chemistry and Physics”, David R. Lide, 84th Edition (2003–2004), CRC Press [34].

**Table 4:** Comparison of the absorption capacities of modified PU foams.

Materials of Absorbent	Modification Approach	^a^ Modification Period	Absorption Capacity (g/g)		Ref
			Oil	Organic Solvent	
Reduced GO/orthoaminophenol/PU foam	Chemical reaction	Long	~33–45	~25–80	[35]
PU-carbon nanotube-polydopamine-octadecylamine foam	Chemical reaction (self-polymerization)	Long	~26–28	~35	[36]
Modified flexible PU foam	Layer by layer (electrostatic deposition)	Long	27–32	20–22	[37]
Magnetic PU foam	Chemical reaction	Moderate	~42–50	~35–77	[38]
Silane functionalized graphene/PU foam	Thermal treatment	Moderate	26.4–44.1	25.8	[39]
PU/PHFBAi	CVD	Short	58.4	53.7–66.7	This study

^a^ The foam modification period (including the time for nanoparticle synthesis) is classified into short (<5 h), moderate (5–20 h), and long (>20 h). Notably, we only considered the articles that have reported the time required to modify the respective foam sample in this table.

## Supplementary Materials

The following are available online at https://www.mdpi.com/2073-4360/12/9/1883/s1, Figure S1: ATR-FTIR spectra of PU/PHFBAi at varying deposition times (zero, 1 min, 5 min, and 10 min) at a spectra range of 1000–1400 cm−1, Table S1: EDX analysis of PU/PHFBAi at varying deposition times.
