# Supplementary material for: A Green Approach to Modify Surface Properties of Polyurethane Foam for Enhanced Oil Absorption
Source: Polymers (Basel). 2020 Aug 21;12(9):1883. doi: 10.3390/polym12091883 (PMC7565495; doi:10.3390/polym12091883)
Supplement: Supplementary file 1 [file polymers-12-01883-s001.pdf]

A Green Approach to Modify Surface Properties of PU Foam for Enhanced Oil Absorption

Supplementary Data

Zhi Chien Ng[1], Rosyiela Azwa Roslan[1], Woei Jye Lau[1,*], Mehmet Gürsoy[2], Mustafa Karaman[2], Nora Jullok[3] and Ahmad Fauzi Ismail[1]

[1]Advanced Membrane Technology Research Centre (AMTEC), School of Chemical and Energy Engineering, Universiti Teknologi Malaysia, 81310, Johor Bahru, Johor, Malaysia
[2]Department of Chemical Engineering, Konya Technical University, Konya, 42075, Turkey
[3] Centre of Excellence for Biomass Utilization, Universiti Malaysia Perlis (UniMAP), Kompleks Pusat Pengajian Jejawi 3, 02600, Jejawi, Perlis, Malaysia

*Corresponding author: lwoeijye@utm.my; lau_woeijye@yahoo.com

This supporting information contains the following:

Figure S1. ATR-FTIR spectra of PU/$PHFBA_i$ at varying deposition time (zero, 1 min, 5 min and 10 min) at spectra range of 1400–1000 cm^{-1}.

Table S1. EDX analysis of PU/$PHFBA_i$ at varying deposition time.

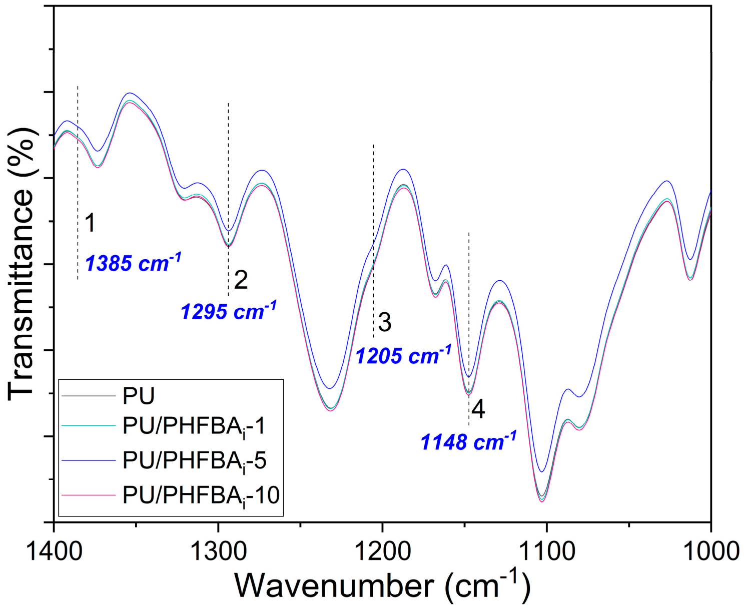

Figure S1. ATR-FTIR spectra of PU/PHFBA$_i$ at varying deposition time (zero, 1 min, 5 min and 10 min) at spectra range of 1400–1000 cm^{-1}.

Table S1. EDX analysis of PU/PHFBA$_i$ at varying deposition time.

Element	Atomic Concentration (at %)		
	PU/PHFBA$_i$-1	PU/PHFBA$_i$-5	PU/PHFBA$_i$-10
C	80.75	82.42	78.59
O	19.02	17.13	20.17
F	0.23	0.45	1.24
